# Comprehensive genetic testing identifies targetable genomic alterations in most patients with non-small cell lung cancer, specifically adenocarcinoma, single institute investigation

**DOI:** 10.18632/oncotarget.7739

**Published:** 2016-02-26

**Authors:** Janani Vigneswaran, Yi-Hung Carol Tan, Septimiu D. Murgu, Brian M. Won, Kathryn Alexa Patton, Victoria M. Villaflor, Philip C. Hoffman, Thomas Hensing, D. Kyle Hogarth, Renuka Malik, Heber MacMahon, Jeffrey Mueller, Cassie A. Simon, Wickii T. Vigneswaran, Christopher H. Wigfield, Mark K. Ferguson, Aliya N. Husain, Everett E. Vokes, Ravi Salgia

**Affiliations:** ^1^ Alpert Medical School, Brown University, Providence, RI, USA; ^2^ Section of Hematology/Oncology, Department of Medicine, The University of Chicago, Chicago, IL, USA; ^3^ Section of Pulmonary and Critical Care Medicine, Department of Medicine, The University of Chicago, Chicago, IL, USA; ^4^ Department of Radiation and Cellular Oncology, The University of Chicago, Chicago, IL, USA; ^5^ Department of Radiology, The University of Chicago, Chicago, IL, USA; ^6^ Department of Pathology, The University of Chicago, Chicago, IL, USA; ^7^ Cancer Registry, Comprehensive Cancer Center, The University of Chicago, Chicago, IL, USA; ^8^ Department of Surgery, The University of Chicago Medicine and Biologic Sciences, Chicago, IL, USA; ^9^ The Dartmouth Institute for Health Policy and Clinical Practice, Lebanon, NH, USA; ^10^ Department of Medical Oncology and Therapeutics Research, City of Hope, Duarte, CA, USA

**Keywords:** non-small cell lung cancer, genetic testing, next-generation sequencing, genomic alteration

## Abstract

This study reviews extensive genetic analysis in advanced non-small cell lung cancer (NSCLC) patients in order to: describe how targetable mutation genes interrelate with the genes identified as variants of unknown significance; assess the percentage of patients with a potentially targetable genetic alterations; evaluate the percentage of patients who had concurrent alterations, previously considered to be mutually exclusive; and characterize the molecular subset of *KRAS*.

Thoracic Oncology Research Program Databases at the University of Chicago provided patient demographics, pathology, and results of genetic testing. 364 patients including 289 adenocarcinoma underwent genotype testing by various platforms such as FoundationOne, Caris Molecular Intelligence, and Response Genetics Inc. For the entire adenocarcinoma cohort, 25% of patients were African Americans; 90% of *KRAS* mutations were detected in smokers, including current and former smokers; 46% of *EGFR* and 61% of *ALK* alterations were detected in never smokers. 99.4% of patients, whose samples were analyzed by next-generation sequencing (NGS), had genetic alterations identified with an average of 10.8 alterations/tumor throughout different tumor subtypes. However, mutations were not mutually exclusive.

NGS in this study identified potentially targetable genetic alterations in the majority of patients tested, detected concurrent alterations and provided information on variants of unknown significance at this time but potentially targetable in the future.

## INTRODUCTION

Personalized medicine using genotyping to inform clinical decisions has become standard of care for non-small cell lung cancer (NSCLC) [1, 2]. Advances in biomarker-driven therapy have markedly improved outcomes for patients with tumor specific molecular abnormalities. Although most national and international guidelines currently recommend testing for *EGFR* mutations and *ALK* gene rearrangements [3–5], it is becoming increasingly clear that more extensive analysis of a tumor's genetic profile is critical to identifying mutations or gene fusions sensitive to already approved therapies but not detected by hotspot testing methods [6]. Next-generation sequencing (NGS) identifies genetic alterations that confer sensitivity to approved and investigational-targeted therapies in patients suffering from a variety of advanced cancers. A previous study showed that a targeted NGS assay identified potentially targetable genetic alterations in 83% of tumors, with 21% of these patients receiving genotype-directed therapy [7]. In that particular study, however, only 7% of patients had lung cancer. High-sensitivity genomic profiling can also reveal potential new pathways to biomarker-driven therapies. In a study of patients with small cell lung cancer who had relapsed after primary chemotherapy, NGS detected at least one targetable alteration with the potential to personalize further therapy in more than 50% of cases [8].

In view of the immediate clinical implications offered by comprehensive molecular testing, we investigated NSCLC patients' genomic profile results of NGS at the University of Chicago for therapeutic purposes. In the past five years, we have collected more than 300 patients' genomic testing and therapeutic results in the University of Chicago Thoracic Oncology database. In this study, we specifically aim to review the results of extensive genetic analysis in patients with NSCLC, specifically adenocarcinoma (AD) from the database. We describe how targetable mutation genes correlate with the genes identified as variants of unknown significance. We assessed the percentage of patients with a potentially targetable genetic alteration. We also evaluated the percentage of patients who had concurrent alterations, previously considered to be mutually exclusive. In addition, we characterize the molecular subset of *KRAS*, one of the most commonly observed genetic mutations in NSCLC AD, for which clinical trials with investigational agents are being conducted [9].

## RESULTS

### Next generation sequencing

One hundred and sixty patient tumor samples were sequenced by FoundationOne from Foundation Medicine (FM) NGS assay over the study period. Table 1 summarizes the demographic characteristics, histology, and molecular markers from specimens analyzed by sending the specimens to Foundation Medicine. African Americans comprised 26% of the study population. Twenty two percent of patients were never smokers. The majority (75%) of patients had adenocarcinoma.

**Table 1:** Summary of the demographic characteristics, histology, and molecular markers from specimens analyzed by FoundationOne

	Total	EGFR	KRAS	ALK	ROS1	RAF1	RET	ERBB2	PIK3CA	MET	CBL	FGF totals
	%(#)	%(#)	%(#)	%(#)	%(#)	%(#)	%(#)	%(#)	%(#)	%(#)	%(#)	%(#)
N =	(160)	(34)	(44)	(11)	(1)	(3)	(16)	(5)	(19)	(11)	(10)	(54)
**Sex**												
Male	49% (79)	41% (14)	39% (17)	45% (5)	0% (−)	67% (2)	56% (9)	60% (3)	74% (14)	64% (7)	40% (4)	52% (28)
Female	51% (81)	59% (20)	61% (27)	55% (6)	100% (1)	33% (1)	44% (7)	40% (2)	26% (5)	36% (4)	60% (6)	48% (26)
**Age**												
Mean	(62.1)	(63.1)	(64.1)	(55.4)	(52.1)	(64.8)	(62.4)	(66.2)	(66.4)	(67.7)	(66.3)	
**Race**												
White	63% (101)	41% (14)	70% (31)	91% (10)	100% (1)	67% (2)	56% (9)	60% (3)	68% (13)	36% (4)	60% (6)	61% (33)
Black	26% (42)	41% (14)	25% (11)	0% (−)	0% (−)	33% (1)	31% (5)	20% (1)	21% (4)	27% (3)	30% (3)	28% (15)
Asian	6% (10)	18% (6)	2% (1)	0% (−)	0% (−)	0% (−)	13% (2)	20% (1)	11% (2)	18% (2)	10% (1)	11% (6)
Other	2% (3)	0% (−)	2% (1)	0% (−)	0% (−)	0% (−)	0% (−)	0% (−)	0% (−)	9% (1)	0% (−)	0% (−)
Unknown	2% (3)	0% (−)	0% (−)	9% (1)	0% (−)	0% (−)	0% (−)	0% (−)	0% (−)	9% (1)	0% (−)	0% (−)
**Smoking**												
Smoker	78% (125)	62% (21)	91% (40)	55% (6)	0% (−)	100% (3)	81% (13)	60% (2)	79% (15)	82% (9)	90% (9)	85% (46)
Never Smoker	22% (35)	38% (13)	9% (4)	45% (5)	100% (1)	0% (−)	19% (3)	40% (2)	21% (4)	18% (2)	10% (1)	15% (8)
**Histology**												
AD	75% (120)	82% (28)	89% (39)	82% (9)	100% (1)	67% (2)	81% (13)	100% (5)	63% (12)	55% (6)	80% (8)	76% (41)
SCC	9% (15)	3% (1)	0% (−)	0% (−)	0% (−)	33% (1)	13% (2)	0% (−)	37% (7)	18% (2)	0% (−)	11% (6)
NSCLC, NOS	4% (7)	3% (1)	2% (1)	9% (1)	0% (−)	0% (−)	0% (−)	0% (−)	0% (−)	9% (1)	10% (1)	6% (3)
LCC	3% (4)	9% (3)	5% (2)	0% (−)	0% (−)	0% (−)	0% (−)	0% (−)	0% (−)	9% (1)	0% (−)	4% (2)
Other	8% (3)	3% (1)	5% (2)	9% (1)	0% (−)	0% (−)	6% (1)	0% (−)	0% (−)	9% (1)	10% (1)	4% (2)

AD: Adenocarcinoma, SCC: Squamous cell carcinoma, LCC: Large cell carcinoma, NOS: Not otherwise specified

### Genetic alterations

**Figure 1:**
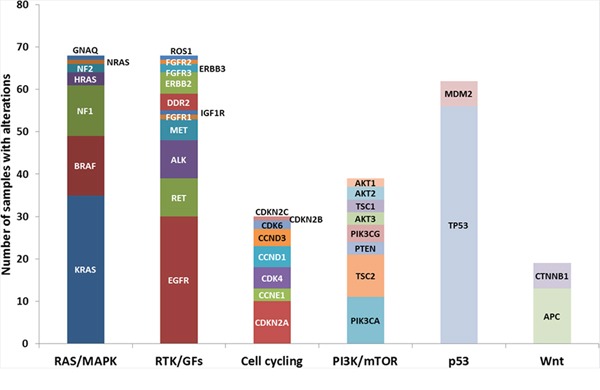
Number of adenocarcinoma samples with genetic alteration classified by cell signaling pathway Genetic alterations from NGS reports were classified by cell signaling pathway, such as RAS/MAPK, RTK/GFs, PI3K/mTOR, p53, Wnt pathways, and cell cycling. The ordinate indicated number of adenocarcinoma samples with alterations. RTK/GFs: Receptor tyrosine kinase/Growth factors.

**Figure 2:**
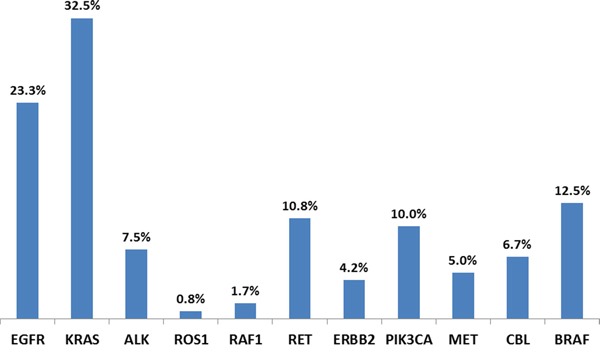
NSCLC adenocarcinoma patients' genetic alterations detected by NGS In all the patient samples tested, 23.3% of *EGFR*, 32.5% of *KRAS*, 7.5% of *ALK*, 0.8% of *ROS1*, 1.7% of *RAF1*, 10.8% of *RET*, 4.2% of *ERBB2*, 10.0% of *PIK3CA*, 5.0% of *MET*, 6.7% of *CBL*, and 12.5% of BRAF alteration were detected.

The vast majority (99.4%) of patients analyzed by NGS had genetic alterations identified with an average of 10.8 alterations/tumor. Targetable gene alterations such as *EGFR, KRAS, ALK, ROS1, RAF1, RET, ERBB2, PIK3CA, MET, FGF,* and *potential targetable gene CBL* were identified throughout tumor subtypes (Table 1). Due to the majority (75%) of tumor samples are AD, we focused our study in only AD populations. The spectrum of potentially targetable genetic alterations including mutations, amplifications, homozygous deletions, and fusions are summarized in Figure 1. Genetic alterations were detected across a wide range of functionally relevant pathways (Fig.1). The most common alterations involved the receptor tyrosine kinase/growth factors (RTK/GFs) genes including *EGFR, ALK, MET, RET* and *ROS1*. Alterations in the mitogen-activated protein kinase (*MAPK*)/*RAS* and phosphatidylinositol 3-kinase (*PI3K*)/*mTOR* pathways were identified in a large proportion of samples with 56.7% (68/120) and 30% (36/120) respectively. Cell cycle-associated genes were dysregulated pathways with mutations, amplifications, or deletions present in 25% (30/120) of tumors. *KRAS* mutation was found in 32.5% of patients with 10% of mutations detected in never smokers (Table 1). Figure 2 summarizes the prevalence of detected alterations in the population studied.

### Concurrent alterations and variants of unknown significance (VUS)

**Figure 3:**
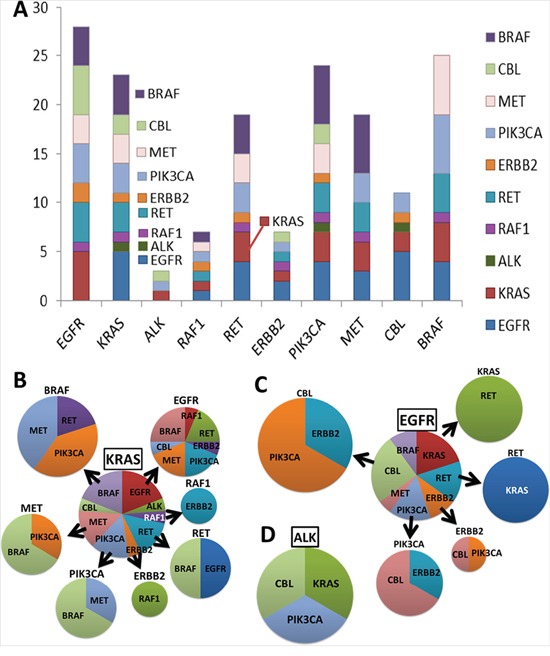
Gene and gene alteration correlation Genetic alteration was detected by NGS. Gene and gene alteration correlation is shown in **A.** A summary of a number of additional mutations (ordinate) coexisting with a specific genetic (abscissa) defect was observed. Samples that had **B.** KRAS, **C.** EGFR, and **D.** ALK were further analyzed respectively. The pie charts show the percentage of samples of each different gene alteration coexisted. Mutations were not mutually exclusive.

We found that not uncommonly, mutations were not exactly mutually exclusive. 36.25% (58/160) of samples had multiple gene alterations including gene amplification. Figure 3A shows the summary along the ordinate the number of additional mutations coexisting with a specific genetic defect. For example, in patients with *KRAS* mutations, additional mutations were detected in *EGFR* (n= 5), *ALK* (n=1), *RAF1* (n=1), *RET* (n=3), *ERBB2* (n=1), *PIK3CA* (n=3), *MET* (n= 3) and *CBL* (n=2). More detailed breakdown of gene and gene alteration correlation of KRAS, EGFR, and ALK are illustrated in Figure 3B, 3C, and 3D. VUS refer to alterations detected in one of the genes included on FoundationOne that have not yet been adequately characterized in the scientific literature. These variants are reported to potentially be acted upon should clinical evidence emerge.

**Figure 4:**
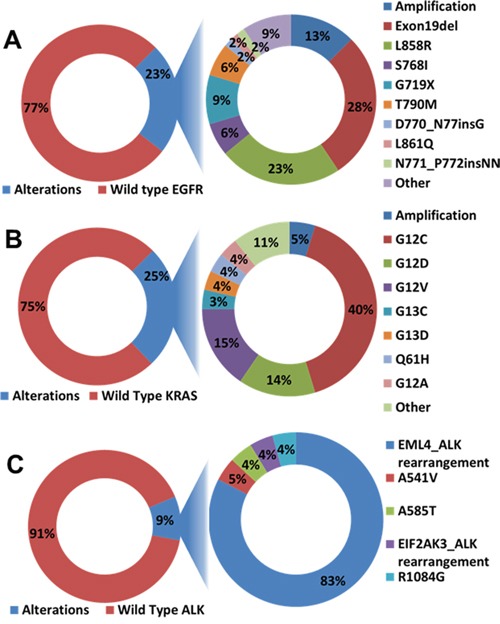
Genomic alteration in the entire cohort of adenocarcinoma samples **A.** *EGFR* alteration. 23% of the *EGFR* tested samples showed genetic alteration. *EGFR* exon 19 deletion and gene amplification showed in majority of the samples. **B.** *KRAS* alteration. 25% of *KRAS* tested samples showed genetic alteration and 40% of these *KRAS* alteration samples had G12C mutation. **C.** *ALK* alteration. 9% of *ALK* tested samples showed genetic alteration and 83% of these *ALK* alteration samples had *EML4-ALK* rearrangements.

Including FM, Caris Molecular Intelligence (CMI), and Response Genetics Inc. (RG) results, the prevalence of genetic alterations, patients and tumor characteristics for the entire AD dataset (N=289) are described in Table 2. African Americans comprised 25% of the study population. Adenocarcinoma was the most commonly tested tumor subtype of NSCLC and NSCLC-NOS comprised 13% of the specimens submitted for molecular analysis. 90% of *KRAS* mutations were detected in smokers, including current and former smokers. 46% of *EGFR* and 61% of *ALK* alteration were detected in never smokers. Thirteen percent of *ALK* alterations were seen in African Americans (Table 2). 85.8% (248/289), 85.5% (247/289), and 85.1% (246/289) of the patients in the entire cohort were tested for *EGFR, KRAS*, and *ALK* respectively. 23% (57/248) of the *EGFR* tested samples showed genetic alteration (Figure 4A). The details of the genetic alteration have been analyzed and are listed in Figure 4. *EGFR* exon 19 deletion showed in a majority of the samples. In addition, 34% (84/247) of *KRAS* tested samples showed genetic alteration and 40.5% (34/84) of these *KRAS* alteration samples had G12C mutation (Figure 4B). 9.3% (23/246) of *ALK* tested samples showed genetic alteration and 78.3% (18/23) of these *ALK* alteration samples had *EML4-ALK* rearrangement (Figure 4C). As in this study, mutations were not mutually exclusive. Some patients with EGFR or KRAS amplification also had other EGFR or KRAS mutations.

**Table 2:** Summary of the demographic characteristics of adenocarcinoma samples from *EGFR, KRAS*, and *ALK* tested specimens analyzed by FoundationOne, Caris Molecular Intelligence, and Response Genetics

	Total	EGFR		EGFR										
	% (#)	Wild Type % (#)	Alteration % (#)	Amplification % (#)	Exon19del % (#)	L858R % (#)	T790M % (#)	L861Q % (#)	S768I % (#)	G719X % (#)	G719A % (#)	D770_N77Ins G % (#)	N771_P772Ins NN % (#)	Other % (#)
N =	(289)	(191)	(57)	(7)	(18)	(15)	(4)	(1)	(3)	(3)	(1)	(1)	(1)	(7)
**Sex**														
Male	40% (116)	41% (78)	33% (19)	29% (2)	11% (2)	47% (7)	50% (2)	100% (1)	0% (−)	0% (−)	0% (−)	100% (1)	0% (−)	57% (4)
Female	60% (173)	59% (113)	67% (38)	71% (5)	89% (16)	53% (8)	50% (2)	0% (−)	100% (3)	100% (3)	100% (1)	0% (−)	100% (1)	43% (3)
**Age**														
Mean	(62.4)	(63.1)	(62.6)	(65.1)	(65.2)	(67.9)	(59.7)	(53.5)	(64.8)	(68.3)	(54.5)	(41.1)	(51.9)	(59.5)
**Race**														
White	65% (187)	70% (134)	42% (24)	43% (3)	33% (6)	33% (5)	75% (3)	100% (1)	100% (3)	0% (−)	100% (1)	100% (1)	0% (−)	71% (5)
Black	25% (73)	23% (43)	39% (22)	57% (4)	56% (10)	13% (2)	0% (−)	0% (−)	0% (−)	100% (3)	0% (−)	0% (−)	100% (1)	29% (2)
Asian	8% (22)	5% (9)	18% (10)	0% (−)	11% (2)	47% (7)	25% (1)	0% (−)	0% (−)	0% (−)	0% (−)	0% (−)	0% (−)	0% (−)
Other	1% (3)	1% (2)	2% (1)	0% (−)	0% (−)	7% (1)	0% (−)	0% (−)	0% (−)	0% (−)	0% (−)	0% (−)	0% (−)	0% (−)
Unknown	1% (3)	1% (2)	0% (−)	0% (−)	0% (−)	0% (−)	0% (−)	0% (−)	0% (−)	0% (−)	0% (−)	0% (−)	0% (−)	0% (−)
**Smoking**														
Smoker	73% (211)	80% (153)	54% (31)	71% (5)	50% (9)	33% (5)	75% (3)	0% (−)	67% (2)	67% (2)	100% (1)	100% (1)	100% (1)	71% (5)
Never Smoker	27% (78)	20% (38)	46% (26)	29% (2)	50% (9)	67% (10)	25% (1)	100% (1)	33% (1)	33% (1)	0% (−)	0% (−)	0% (−)	29% (2)

	Total	KRAS		KRAS								
	% (#)	Wild Type % (#)	Alteration % (#)	Amplification % (#)	G12C % (#)	G12D % (#)	G12V % (#)	G12A % (#)	G13C % (#)	G13D % (#)	Q61H % (#)	Other % (#)
N =	(289)	(163)	(84)	(5)	(34)	(12)	(13)	(3)	(3)	(3)	(3)	(8)
**Sex**												
Male	40% (116)	38% (62)	38% (32)	20% (1)	50% (17)	25% (3)	8% (1)	33% (1)	67% (2)	67% (2)	67% (2)	38% (3)
Female	60% (173)	62% (101)	62% (52)	80% (4)	50% (17)	75% (9)	92% (12)	67% (2)	33% (1)	33% (1)	33% (1)	63% (5)
**Age**												
Mean	(62.4)	(62.4)	(63.6)	(77.1)	(62.8)	(65.5)	(62.8)	(72.2)	(65.8)	(68.4)	(65.8)	(59.9)
**Race**												
White	65% (187)	60% (98)	69% (58)	80% (4)	65% (22)	75% (9)	69% 9	67% (2)	100% (3)	33% (1)	67% (2)	75% (6)
Black	25% (73)	26% (42)	27% (23)	20% (1)	32% (11)	25% (3)	23% (3)	33% (1)	0% (−)	67% (2)	0% (−)	25% (2)
Asian	8% (22)	11% (18)	2% (2)	0% (−)	3% (1)	0% (−)	8% (1)	0% (−)	0% (−)	0% (−)	0% (−)	0% (−)
Other	1% (3)	1% (2)	1% (1)	0% (−)	0% (−)	0% (−)	0% (−)	0% (−)	0% (−)	0% (−)	33% (1)	0% (−)
Unknown	1% (3)	1% (2)	0% (−)	0% (−)	0% (−)	0% (−)	0% (−)	0% (−)	0% (−)	0% (−)	0% (−)	0% (−)
**Smoking**												
Smoker	73% (211)	67% (109)	90% (76)	60% (3)	100% (34)	92% (11)	69% (9)	100% (3)	100% (3)	100% (3)	100% (3)	88% (7)
Never Smoker	27% (78)	33% (54)	10% (8)	40% (2)	0% (−)	8% (1)	31% (4)	0% (−)	0% (−)	0% (−)	0% (−)	13% (1)

	Total	ALK		ALK				
	% (#)	Wild Type % (#)	Alteration % (#)	EML4_ALK % (#)	A541V % (#)	A585T % (#)	EIF2AK3_ALK	R1084G % (#)
N =	(289)	(223)	(23)	(19)	(1)	(1)	(1)	(1)
**Sex**								
Male	40% (116)	38% (84)	52% (12)	58% (11)	0% (−)	0% (−)	0% (−)	100% (1)
Female	60% (173)	62% (139)	48% (11)	37% (7)	100% (1)	100% (1)	100% (1)	0% (−)
**Age**								
Mean	(62.4)	(63.5)	(50.5)	(48.8)	(69.3)	(47.8)	(71.6)	(63.8)
**Race**								
White	65% (187)	61% (135)	87% (20)	79% (15)	100% (1)	100% (1)	100% (1)	100% (1)
Black	25% (73)	28% (63)	13% (3)	16% (3)	0% (−)	0% (−)	0% (−)	0% (−)
Asian	8% (22)	9% (20)	0% (−)	0% (−)	0% (−)	0% (−)	0% (−)	0% (−)
Other	1% (3)	1% (2)	0% (−)	0% (−)	0% (−)	0% (−)	0% (−)	0% (−)
Unknown	1% (3)	1% (2)	0% (−)	0% (−)	0% (−)	0% (−)	0% (−)	0% (−)
**Smoking**								
Smoker	73% (211)	76% (169)	39% (9)	26% (5)	100% (1)	100% (1)	0% (−)	100% (1)
Never Smoker	27% (78)	24% (54)	61% (14)	68% (13)	0% (−)	0% (−)	100% (1)	0% (−)

## DISCUSSION

We report a single institution's 5-year experience of implementing comprehensive genetic testing including NGS in patients with advanced NSCLC. Genetic alterations were identified by NGS in the vast majority of patients with NSCLC, and this analysis shows the changing platforms that have occurred over the past several years. Currently targetable alterations were identified in a significant proportion of NSCLC. Our findings highlight the importance of testing not only adenocarcinoma, but to broaden the testing to the other NSCLC subtypes as is currently recommended by guidelines [10]. The variety of genetic alterations reported independent of race, age, sex or smoking status is also in line with the current recommendations from national and international guidelines [10–12].

A prior study showed that the presence of KRAS mutation excludes the possibility of *EGFR* mutation or *ALK* translocation [13]. In fact, some testing algorithms suggested to first test for *KRAS* and if negative proceed with *EGFR*, then *ALK* [14]. Coexisting targetable alterations were indeed not uncommon in our patients. Our findings would suggest that we should have a more comprehensive approach rather than a sequential approach. Given the turnaround time of at least 2-3 weeks (and longer if a patient has the biopsy while hospitalized due to Medicare/insurance rules) and the advanced disease state of this patient population, sequential testing (even when only *KRAS, EGFR* and *ALK* status are analyzed) would delay care. Targeted NGS approach has potential value in several additional ways. Novel, potentially active therapies can be identified, enabling clinical trial enrollment for patients without available treatment options. Even “negative” results from NGS are useful to direct patients toward immunotherapy (anti-PD1, anti-PD-L1) trials, further chemotherapy or comfort care. NGS allows identifications of VUS, which could become valuable in the near future as our understanding of prognostic and predictor markers of response continues to evolve. Oncogenic fusions, such as those involving kinases, continue to be discovered (i.e. *KIF5B-RET*) and may have immediate implications on lung cancer management [15].

There are several limitations to this study. Results were evaluated in a retrospective fashion. The impact of NGS on downstream cost-effectiveness was not thoroughly assessed in this analysis. Published data suggest, however, that at least one out of five patients tested by NGS receives biomarker-driven treatment [7].

A major challenge to routine implementation in the care of patients with advanced NSCLC relates to tissue requirements for NGS. Our study did not evaluate the tissue requirements for NGS testing as part of a retrospective analysis because it was impossible to collect accurate data on the volume and cellularity of acquired specimens submitted for molecular analysis. At this time, comprehensive molecular analysis requires adequacy of tumor biopsy material. For histology specimens, for instance, FFPE samples stored as either tissue blocks or in unstained slides are required. A total tumor volume of > 1mm^3^ with > 80% cellularity (or > 30,000 cells) and tumor content (ratio of malignant to nonmalignant cells) of >20% are required for some targeted NGS assays [7]. However, approximately 70% of lung cancer patients are diagnosed and staged using small biopsy specimens or cytology specimens [16]. It is unclear how many biopsies (transbronchial, endobronchial or transthoracic) are necessary to assure adequate amount for sample for testing. At the time of this writing, a 400-gene panel requires approximately 60 ng of DNA while a 50-gene panel, approximately 10 ng of DNA. As one cell contains approximately 1 pg DNA, it results that the number of cells needed for current NGS panels varies between 10 and 60,000 cells. It is thus relevant for the proceduralist to acquire as much target tissue as safely possible and for the pathologist to minimize the number of immunostains (i.e. limit to P40/P63 and TTF-1), carefully review the specimens and quantify the cellularity prior to submission for NGS testing. Tissue requirement challenges may be soon overcome by blood-based assays. Circulating tumor cells and cell-free tumor DNA have shown promise for non-invasive genomic profiling to guide targeted therapy in NSCLC. Advancements in molecular technology including various isolation strategies and cell separation techniques could soon make it possible to routinely analyze clinically targetable genetic drivers in blood.

Based on current guideline recommendations [10, 17, 18] patients should undergo diagnosis and staging in the same procedure. This is often achieved though endobronchial ultrasound-guided transbronchial needle aspiration (EBUS-TBNA) from mediastinal lymph nodes. It remains to be determined whether EBUS-TBNA specimens (smears or cell blocks) are adequate for comprehensive NGS testing. NGS was shown, however, to be feasible in fine-needle aspiration biopsy (FNAB, FNA or NAB) cytology specimens from pulmonary and pancreatic lesions [19]. Molecular profiling of cytology samples has been shown to be reliable when compared with histological samples from the same patient [20, 21]. The use of smears for molecular testing is feasible and in fact could expedite care as smears can be reviewed as part of the rapid on site cytological examination (ROSE) at the time of the FNA (EBUS-, EUS- or CT-guided). Indeed, the addition of ROSE improves the adequacy of EBUS-TBNA specimens for molecular profiling (EGFR, KRAS and ALK) and prevents the need for a repeat invasive diagnostic procedure aimed at molecular testing in at least 1 out of 10 patients [22]. To date, however, we don't have enough evidence to show the relative success rates between core biopsies and needle aspirations techniques. In addition, core biopsies revealing tissue architecture may provide a better appreciation of the tumor-stroma and tumor-immune cells relationship, relevant for biomarker testing in immune-oncology. Whether this information can be obtained via needle techniques remains to be determined.

There is a minimum number of genes required for testing in advanced NSCLC. In one study, the authors tested at least eleven genes for potential implication in adenocarcinoma [23] and demonstrated that in non-smoking adenocarcinomas, there can be other alterations in a larger NGS panel. NGS identified targetable genomic alterations in 65% of tumors from patients with lung cancer who never smoked or were light smokers, whose tumors were deemed without targetable genomic alterations by earlier extensive non-NGS testing. These findings support first-line profiling of lung adenocarcinomas using an NGS approach as a more comprehensive and efficient strategy compared to non-NGS testing.

In conclusion, this study of targeted NGS in a group of patients with NSCLC cancer identified potentially targetable genetic alterations in the majority of patients across various tumor histology subtypes. NGS provides additional information by uncovering targetable concurrent alterations or alteration of unknown significance at this point in time, but potentially targetable in the future. The immediate consequences of NGS are relevant as genotype-directed treatment options for patients can be promptly implemented and enrollment in clinical trials can be expedited. NGS will likely continue to increase in importance as molecularly targeted therapeutic agents continue to show efficacy. The exact nature, quantity, and quality of specimens submitted for such comprehensive molecular analysis remains to be determined.

## MATERIALS AND METHODS

### NSCLC samples and subjects

Patients suffering from advanced NSCLC (n=364), specifically adenocarcinoma (n=289), included in this analysis were evaluated at the University of Chicago Hospitals from December 2009 to August 2014 and underwent genotype testing at the discretion of the primary clinical provider. Patients were consented to Institutional Review Board (IRB) approved protocols 9571 and 13473A or added under protocol 10-654N for deceased patients. This permitted the research team to access medical records for chart abstraction as well as analysis of results of clinical genetic testing for retrospective study. Tissue retrieved by core needle biopsies, excisional biopsies, or surgical resection could undergo NGS. Formalin-fixed paraffin-embedded (FFPE) samples stored as either tissue blocks or in unstained slides were procured by the testing facility. FoundationOne (Foundation Medicine, FM; Cambridge, MA), Caris Molecular Intelligence (CMI; Irving, TX), and Response Genetics Inc. (RG; Los Angeles, CA) were the companies that performed the genetic testing. Patients with genetic testing ordered from multiple companies done on the same specimen (n=3) had genetic results combined, utilizing the result from the company that performed the test. Patients with multiple biopsies sent for genetic testing were included as separate entries (n = 22).

### Databases and study design

Retrospective review of Thoracic Oncology Research Program (TORP) Microsoft Access Databases provided patient demographics, pathology, and results of genetic testing. The TORP Access database has been previously described and validated [24–26]. Manual data abstraction from the patient files on electronic medical records (EPIC) was also performed for missing fields. The Cancer Registry database was utilized to obtain current vital status as well as date of death not recorded in Epic. Patients with a histologically confirmed diagnosis of NSCLC were included in this study if molecular testing was performed on their tumor. There were no restrictions of tumor histology, disease stage, subsequent or previous treatment or performance status. All the analyses were performed in Excel, with basic functions and formulas.

### Genotype testing

The targeted NGS assays developed by FoundationOne from FM has been previously described and validated [27]. Samples sent to FM underwent whole-genome shotgun library construction with hybridization–based capture for 4,557 exons from 287 cancer related genes and 47 introns from 19 genes frequently involved in DNA rearrangements. Samples sent to CMI underwent an established technology platforms to measure a panel of carefully selected biomarkers including immunohistochemistry (IHC), fluorescent in situ hybridization (FISH), polymerase chain reaction (PCR), and direct gene sequencing [28]. RG paired NGS with FISH and RNA expression markers to identify any clinically targetable genetic alteration in the samples. This technique has also been previously validated [29]. The reports were focused on FoundationOne NGS due to its complete panel of gene alteration testing in comparison with CMI and RG. Clinical testing for most genetic variants is performed in a CLIA-certified molecular genetics or molecular pathology laboratory. Our focus was on NGS, not on IHC or FISH, although we report the ALK FISH testing because of its accepted relevance to therapeutic decision making.

### Statistical considerations

No formal statistical hypotheses were assessed. Statistical analyses were descriptive. The number of patients with genetic testing information determined the sample size. All descriptive analysis was performed using frequencies and percentiles.
